# The Medical Graduates' College and Polyclinic

**Published:** 1907-11-23

**Authors:** 


### THE MEDICAL GRADUATES' COLLEGE AND POLYCLINIC.

Professor Clifford Allbutt's lecture last week on "The Senile Cardio-Vascular System" was specially written and elaborated from the reports of a lecture delivered at the Medical Graduates' College and Polyclinic. The lecture in question was one of a series of addresses on matters connected with senility which has been arranged for this session. Those which have already been delivered include "The Administration of Anæsthetics to the Aged," by Dr. F. W. Hewitt; "The Psychoses of the Aged," by Dr. G. H. Savage; that by Professor Clifford Allbutt above referred to; "The Eyesight in Old Age," by Mr. Marcus Gunn; and "Senile Respiratory Disorders," by Sir Richard Douglas Powell. There remain to be delivered "Diet in Old Age," by Dr. Burney Yeo (December 4), and "The Skin Troubles of the Aged," by Dr. Ratcliffe Crocker (December 12).

